# *Farmácia Popular* Program: changes in geographic accessibility of medicines during ten years of a medicine subsidy policy in Brazil

**DOI:** 10.1186/s40545-015-0030-x

**Published:** 2015-03-09

**Authors:** Isabel Cristina Martins Emmerick, José Miguel do Nascimento, Marco Aurélio Pereira, Vera Lucia Luiza, Dennis Ross-Degnan, ISAUM-Br Collaborative Group

**Affiliations:** Department of Population Medicine, Harvard Medical School and Harvard Pilgrim Health Care Institute, 133 Brookline Avenue, 6th Floor, Boston, MA 02215 USA; Departamento de Assistência Farmacêutica/Secretaria de Ciência Tecnologia e Insumos estratégicos – Ministério da Saúde – Brasil - DAF/SCTIE/MS Esplanada dos Ministérios, Bloco G, 8º andar, CEP: 70058-900 Brasília, DF Brazil; Nucleus for Pharmaceutical Policies, National School of Public Health, Oswaldo Cruz Foundation, 1480, Rua Leopoldo Bulhões # 624, Manguinhos, 21021-000 Rio de Janeiro, RJ Brazil

**Keywords:** Government programs, Brazil, Essential medicines, Access, Co-payment

## Abstract

**Objectives:**

The Brazilian constitution guarantees the right to health, including access to medicines. In May 2004, Brazil’s government announced the *“Farmácia Popular”* Program (FPP) as a new mechanism to improve the Brazilian population’s access to medicines. Under FPP, a selected list of medicines is subsidized by the government and provided in public and private pharmacies.

The aim of this study is to describe the historical stages of the FPP and to identify associated changes in the geographical accessibility of medicines through the FPP over time.

**Methods:**

It was performed documentary review and an ecological study assessing program coverage in terms of number of facilities and a FPP Pharmacy Facilities Density (PFD) index at national and regional levels from 2004 to 2013, using the FPP database. We used geographic information system mapping to depict a pharmaceutical facilities density (PFD) index at the municipality level on thematic maps.

**Results:**

A growth of the PFD index coincident with the phases of the FPP was noticed. In the public sector, the program started in 2004; by 2006, there was a sharp increase in the numbers of participating pharmacies, stabilizing in 2009. In the private sector, the program started in 2006; by 2009 the PFD ratio had increased substantially and it continued to grow through 2011. There was an increase in FPP coverage in most regions between 2006, when the private pharmacy component started, and 2013, but participating pharmacies remain unequally distributed across geographical regions. Specifically, the wealthy areas in the South and Southeast have higher coverage, with lower coverage mostly in the North and Northeast, relatively poorer areas with greater need for access to medicines, health care, and other basic services such as potable water and sanitization.

**Conclusions:**

There has been a substantial increase in the number of pharmacies participating in the FPP over time. This has led to greater program coverage and has potentially improved access to FPP medicines in the country. Nevertheless, disparities in pharmacy coverage remain among the regions.

**Electronic supplementary material:**

The online version of this article (doi:10.1186/s40545-015-0030-x) contains supplementary material, which is available to authorized users.

## Introduction

Equitable access to health care and medicines is a challenge worldwide. The World Health Organization (WHO) considers equitable access to safe and affordable medicines as vital to achieving the highest possible standard of health for all [1]. In the Latin America and Caribbean (LAC) region, health expenditures are estimated to account for more than one-third of all household expenditures [2], of which a large proportion is dedicated to medicines. The high prices of medicines and the need for high out-of-pocket payments by patients represent important barriers in their access [3].

A government subsidy system is one method to expand access to medicines in a sustainable way. In Brazil, total expenditures on health services accounted for 5.5% of the Gross Domestic Product (GDP) and total expenditures on medicines accounted 1.9% of GDP in [4]. Data from the most recent household expenditures survey shows that health is the fourth most important expenditure category, after housing, food and transportation; medicines accounted for about 47% of total household expenditures on health, with higher expenditure burden among the poorest [5].

Non-communicable diseases (NCDs) are the leading causes of death in the world, responsible for 63% of the 57 million deaths that occurred in 2008. The majority of these deaths, were attributed to cardiovascular diseases, diabetes, cancers, and chronic respiratory diseases [6]. NCDs are also the leading causes of premature death and illness throughout the Americas [7]. In Brazil, 72% of deaths in 2007 are attributed to NCDs, with heart disease being the leading cause [8,9]. About 12.2% of all hospitalizations not related to pregnancies and 15.4% of all hospital costs in the period 2008-2010 could be attributed to diabetes. Control of NCDs continues to be one of top health priorities in Brazil, addressed through a set of integrated policies [8]. Access to and appropriate use of medicines are crucial for controlling NCDs, especially hypertension and diabetes, contributing to improved health outcomes and quality of life.

Governments or third-party payers subsidize medicines when they pay a percentage of the cost, with patients responsible for the remained. One important question that arises regarding the effect of medicine subsidies is whether subsidies increase overall access to medicines for all population segments. Currently, there are few studies conducted in LMIC that attempt to address this critical question. Most existing studies have weak designs and limited analytic scope. Lack of knowledge about the effectiveness of subsidy policies in low and middle-income countries (LMICs) presents a barrier to [10].

In Brazil, access to health care, including access to medicines, is a citizen’s constitutional right and the government’s responsibility. The Brazilian health system, known as SUS (Unified Health System–“*Sistema Único de Saúde”*), is organized by the principles of universal coverage, management decentralization, health assistance integrity, and community participation [11].

The National Health System consists of a tax-funded public sector, where care is offered free of charge to the entire population, and a private sector, comprising diverse prepayment mechanisms such as health insurance and out-of-pocket financing. Private sector health facilities and practitioners also provide health services under contracts with the government [12].

Before 2004, medicines in Brazil were obtained through two pathways, either free in public health care facilities or paid out-of-pocket in the private sector (retail pharmacies). In May 2004, Brazil’s government announced an additional mechanism to improve the Brazilian population’s access to medicines [13]. The policy called *“Farmácia Popular do Brasil”* Program (FPP) specified a list of essential medicines to be subsided initially in by the government and supplied in public pharmacies; some years later, this program was expanded to contracted private pharmacies.

Brazil has historically exhibited important regional socioeconomic and health system disparities (Table 1) in terms of social indicators and urban infrastructure [14]. The lower reported prevalence of hypertension and diabetes may be attributed in part to lower diagnostic capacity.

**Table 1:** **Economic, health structure and health indicators, Brazil and regions, 2006 to 2012**

	**2006**	**2007**	**2008**	**2009**	**2010**	**2011**	**2012**
GDP per capita(USD)*							
Brazil	5,824	7,215	8,715	8,470	11,229	12,856	-
North	3,667	4,469	5,568	5,320	7,215	8,291	-
Northeast	2,767	3,421	4,081	4,089	5,432	6,196	-
Southeast	7,764	9,556	11,544	11,088	14,763	16,925	-
South	6,499	8,224	9,950	9,675	12,908	14,556	-
West-Center	7,137	8,962	11,117	11,197	14,175	16,614	-
Life expectancy on birth (years)							
Brazil	72.4	72.5	72.8	73.1	73.4	74.2	75.4
North	71.3	71.6	71.9	72.2	72.4	71.1	71.3
Northeast	69.4	69.7	70.1	70.4	70.8	71.6	71.9
Southeast	73.8	74.1	74.3	74.6	74.9	75.9	76.3
South	74.4	74.7	75.0	75.2	75.5	76.2	76.6
West-Center	73.5	73.7	74.0	74.3	74.5	77.5	77.7
Number of MD per inhabitant**							
Brazil	1.3	1.3	1.4	1.5	1.5	1.5	1.6
North	0.6	0.7	0.8	0.8	0.8	0.8	0.8
Northeast	0.8	0.9	0.9	1.0	1.0	1.0	1.0
Southeast	1.6	1.7	1.8	1.9	2.0	2.0	2.1
South	1.3	1.4	1.5	1.6	1.7	1.6	1.7
West-Center	1.3	1.3	1.5	1.5	1.6	1.6	1.6
Diabetes prevalence per 100 inhabitants							
Brazil	8.8	8.8	9.7	9.5	9.9	10.3	11.7
North	8.6	7.6	7.2	7.5	8.4	9.5	8.4
Northeast	8.5	9	9.5	9.6	9.1	10.3	10.7
Southeast	9.3	9.3	10.6	10.1	10.7	10.7	12.9
South	7.9	8.7	8.5	9.3	10.5	9.4	12.5
West-Center	7.8	7.2	8.4	8.1	9.1	9.4	10.5
Hypertension prevalence per 100 inhabitants (Population 25 years old and over)							
Brazil	21.5	22.3	23.9	24.4	23.3	24.3	24.3
North	19.1	17.3	17.4	18.9	18.1	19.9	18.7
Northeast	21.4	21.6	22.3	23.5	22.0	23.2	23.9
Southeast	22.8	24.6	27.0	26.8	25.2	26.2	25.8
South	20.9	21.8	23.3	22.8	23.8	24.4	24.7
West-Center	19.4	19.6	20.8	22.3	22.6	23.2	24.1

Source: Basic data and indicators - Brazil - 2012 IDB-2012 – Department of Informatics of the National Health System (DATASUS) available in: <http://tabnet.datasus.gov.br/cgi/idb2012/matriz.htm>

*regional data is not available for 2012;

**Medical Doctors registered in the national database of health establishments.

Marked geographic inequalities in access to health services and health outcomes are also present; while the prevalence of morbidity is inversely proportional to household income per capita and thus higher in the North and Northeast, the rate of health services use in those regions is lower [15].

This paper was developed under a broader study denominated Impact of consecutive subsidies policies on access to and use of medicines in Brazil (ISAUM-Br Project) [16,17]. The main project goal is to evaluate the impact Brazilian subsidies policies *“Farmácia Popular”* Program (FPP) in its four consecutive phases on access and use of medicines.

This paper describes the *“Farmácia Popular”* Program (FPP) in its four consecutive phases and the changes in coverage and geographic scope of the program that have occurred over time. Understanding the geographic impacts of these changes in Brazilian medicines subsidy policy will increase knowledge about whether large government subsidies for specific categories of medications can reduce disparities in access.

## Methods

It was performed a documentary review of the FPP from 2004 to 2013 and an ecological study that assessed coverage in terms of the number of participating facilities and a Pharmacy Facilities Density (PFD) index at national and regional levels, using data from the FPP and the Ministry of Health.

The documentary review intended to describe the FPP and its stages of implementation according to official regulations, including technical changes in the program. We performed a search of documents in *"Saúde Legis"*–a database containing all legislation related to health in Brazil-and the Brazilian Ministry of Health website, that contains all technical guidelines formulated at the federal level. The keywords used were *“farmácia popular”* or “farmacia” AND “popular” from January 2004 to January 2013. All documents concerning “*Farmacia Popular”* regulations were included, and exclusion criteria do not apply.

In order to identify changes in geographical coverage of the FPP program over time, three indicators were used: number of facilities; percentage of municipalities covered (i.e., municipalities with one or more pharmacies in the FPP); and Pharmacy Facilities Density (PFD, number of pharmacies affiliated with the FPP per 100,000 inhabitants). These measures were applied separately for public and private pharmacies. A growth index was calculated summarizing the percentage change over time, considering 2006 as baseline year for comparison, since the number of facilities in 2004 on the different stratum are zero. When facilities did not exist in a specific population stratum in 2006, we used 2008 as the base year. The selected years correspond to the years before and after each *"Farmácia Popular"* intervention.

These indicators were calculated for the population of Brazil as a whole, for each region, and by municipality size, which was considered as a proxy for urban/rural, considering small municipalities (rural) as those with 20,000 inhabitants or less and medium/large municipalities (urban) as those with greater than 20,000 inhabitants. Additionally, geographic information system mapping was used [18] to allow visualization of the PFD ratio at municipality level on thematic maps for the years 2006 and 2013.

The ISAUM-Br project [17] was reviewed and approved by the World Health Organization Research Ethics Review Committee (WHO – ERC) under the protocol identification number RPC554 and the Brazilian National Ethical Committee under the protocol number 438.743.

## Results

In the literature review, 211 relevant documents were found; 30 documents in the Ministry of Health website and 176 in the “*Saúde Legis”*. (Complete results of the documents search in Additional file 1).

**Figure 1:**
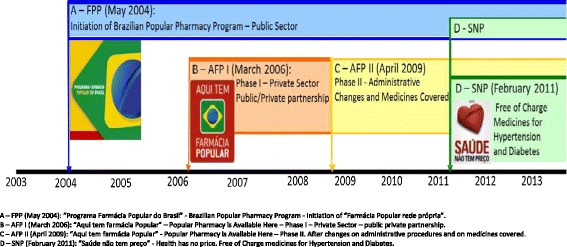
**Time line of the** ***"Farmácia Popular"*** **Program. A**–FPP (May 2004): *“Programa Farmácia Popular do Brasil”*-Brazilian Popular Pharmacy Program-Initiation of *“Farmácia Popular rede própria”*. **B**–AFP I (March 2006): *“Aqui tem farmácia Popular”*–Popular Pharmacy is Available Here–Phase I–Private Sector–public private partnership. **C**–AFP II (April 2009): *“Aqui tem farmácia Popular”*-Popular Pharmacy is Available Here–Phase II. After changes on administrative procedures and on medicines covered. **D**–SNP (February 2011): *“Saúde não tem preço”*-Health has no price. Free of Charge medicines for Hypertension and Diabetes.

The *“Farmácia Popular”* Program (FPP) was created in 2004 and during the succeeding 10 years the FPP experienced three main changes (Figure 1). In 2006, the government expanded the program to the private retail pharmacies; in 2009 the medicines list (Additional file 2) was expanded and some administrative requirements changed; and in 2011, medicines for diabetes and hypertension started to be fully subsidized. The medicines list covered also changed over time, becoming broader and covering more diseases. Three modalities of *"Farmácia Popular"* are concurrently in place at this time: FPP in public facilities; AFP in accredited private retail pharmacies; and SNP which covers a subset of medicines targeting relevant chronic diseases that are dispensed to patients with no co-payment in both the FPP and AFP. These modalities are described below.

In 2004, Brazil’s government announced *“Farmácia Popular do Brasil”* (FPP) a new mechanism to improve the Brazilian population’s access to medicines [13]. This policy specified a list of medicines to be subsided by the government and supplied in public pharmacies and it was especially aimed at low income people covered by private health care insurance, since in Brazil, few private insurance programs include outpatient medicines as a benefit [19]. Considering the size of Brazil, the total number of pharmacies was low, especially in the north and northeast regions.

The FPP, initiated in May 2004 involved a public network of facilities such as university hospitals and NGOs that were coordinated by the Oswaldo Cruz Foundation (Fiocruz) on behalf of the Ministry of Health (MoH) and developed through partnerships with states and municipalities [13]. The project to install a *“Farmácia Popular”* facility is standardized and includes minimum infrastructure requirements, human resources (quantity, qualification, and uniform), and equipment. The federal government, through Fiocruz management, is responsible for funding infrastructure and maintenance, including training, employee payment, and purchase of medicines [19]. The sale price of medicines, which means the price paid by the patients, in these pharmacies is established by the federal government and comprises the medicine value, purchased through open bidding, plus the pharmacy operating costs [19].

In March 2006, the government expanded this policy to include retail pharmacies in the private sector [20]. Under this policy, 90% of the reference price of a limited list of medicines would be subsided by the government in private pharmacies, with the remaining paid by the consumer [21]. Prices paid by patients varied depending on the relation between the reference price (RP) (established by the MoH for each medicine) and its selling price (SP). If the SP is lower than the RP, the government pays 90% of the SP. If the SP is equal to or greater than the RP, the government pays 90% of the reference value [17].

The AFP Phase I was implemented through partnerships with private retail pharmacies, but with a limited set of the medicines than available in the public sector program [21]. This allowed rapid expansion of the number of establishments, enabling wider program coverage, from around 3,000 pharmacies in 2006 to 6,500 in 2008 (Table 2). The management is carried out directly by the Department of Pharmaceutical Services/Office of Science Technology and Strategic Resources-Ministry of Health (*Departamento de Assistência Farmacêutica/Secretaria de Ciência Tecnologia e Insumos estratégicos–Ministério da Saúde–Brasil-DAF/SCTIE/MS*). The minimum requirements for the establishments, which included sanitary authorization of operation, presence of a technically responsible pharmacist, fiscal capability, and infrastructure for a computerized system) were requirements for accreditation of the participating private pharmacies [21].

**Table 2:** **Number of** ***“Farmácia Popular“*** **Facilities in public and private sectors, Brazil and regions, 2004 to 2013**

	**2004**	**2005**	**2006**	**2007**	**2008**	**2009**	**2010**	**2011**	**2012**	**2013**
Public sector facilities										
Brazil	27	75	259	407	504	530	543	555	558	558
North	0	5	24	55	68	73	76	75	75	75
Northeast	7	21	91	137	172	177	182	188	192	192
Southeast	18	40	97	147	176	188	188	193	194	194
South	1	5	35	48	60	62	65	66	65	65
West-Center	1	4	12	20	28	30	32	33	32	32
Private sector facilities										
Brazil			2,955	5,052	6,459	10,790	14,000	20,165	25,120	25,150
North			90	124	137	169	359	587	693	693
Northeast			351	535	619	780	1,027	1,839	2,758	2,766
Southeast			1,746	2,916	3,700	6,416	7,742	10,291	12,423	12,443
South			581	1,157	1,592	2,780	3,857	5,673	6,824	6,833
West-Center			187	320	411	645	1,015	1,775	2,422	2,415

In April 2009, the government expanded the list of medicines while retaining the same degree of subsidy as FPP under a program named *“Aqui Tem Farmácia Popular”* phase II (AFP–Phase II) [22]. The expanded list contains medicines for hypertension, diabetes, and contraception [23]. In 2010, the list was further broadened to include medicines for rhinitis, asthma, Parkinson disease, osteoporosis, glaucoma, and adult diapers [24]. In the AFP Phase II, reorganization included changes in the methodology for accreditation, changes in the computerized system, and greater accuracy in MoH payment to retail pharmacies [22]. The number of establishments reached 25,120 in 2012, covering 63.4% of Brazilian municipalities, but with higher coverage in the medium/large municipalities (84.0%) compared to small municipalities (54.6%) (Table 3).

**Table 3:** **Percentage of municipalities covered by the** ***“Aqui tem Farmácia Popular“*** **Program (AFP) (at least one private pharmacy) overall and by small (20,000 inhabitants and under) and Medium/Large (greater than 20,000 inhabitants) municipalities, Brazil and regions, 2006 to 2012**

	**2006**	**2008**		**2010**		**2012**	
**All municipalities**	**%coverage**	**%coverage (Growth index)**		**%coverage (Growth index)**		**%coverage (Growth index)**	
Brazil	7.0%	19.4%	(2.78)	40.5%	(5.80)	63.4%	(9.08)
North	2.2%	5.3%	(2.40)	12.7%	(5.70)	29.1%	(13.10)
Northeast	2.5%	6.6%	(2.68)	13.1%	(5.34)	41.2%	(16.80)
Southeast	13.2%	34.6%	(2.62)	67.2%	(5.10)	84.1%	(6.37)
South	9.0%	27.1%	(3.02)	58.2%	(6.48)	80.9%	(9.00)
West-Center	1.7%	8.6%	(5.00)	32.5%	(19.00)	63.8%	(37.25)
**Medium/large municipalities (greater than 20,000 inhabitants)**							
Brazil	21.7%	46.1%	(2.12)	63.9%	(2.95)	84.0%	(3.87)
North	5.7%	13.1%	(2.30)	24.4%	(4.30)	49.4%	(8.70)
Northeast	7.3%	18.1%	(2.48)	30.9%	(4.23)	71.7%	(9.80)
Southeast	38.8%	76.1%	(1.96)	94.3%	(2.43)	99.2%	(2.56)
South	37.5%	79.8%	(2.13)	96.8%	(2.58)	99.2%	(2.64)
West-Center	7.2%	29.7%	(4.13)	85.6%	(11.88)	98.2%	(13.63)
**Small municipalities (20,000 inhabitants and under)**							
Brazil	0.7%	8.0%	(11.59)	30.5%	(44.11)	54.6%	(78.93)
North	0.0%	0.4%	NA	5.1%	(14.00)	16.1%	(44.00)
Northeast	0.0%	0.8%	NA	4.1%	(5.44)	25.8%	(34.22)
Southeast	1.3%	15.4%	(11.67)	54.6%	(41.53)	77.0%	(58.53)
South	1.3%	12.9%	(10.08)	47.8%	(37.33)	75.9%	(59.33)
West-Center	0.0%	2.0%	NA	16.0%	(8.14)	53.1%	(27.00)

Advertising of the program is standardized by the government and visual inspection of the retail pharmacy facilities is mandatory [25]. Display of an easily visible document (such as a chart) containing the medicines list and corresponding price list is required for pharmacies in the program [26]. During these successive policies, a physician prescription has always been required for dispensing even for OTC medicines.

The “Saúde não tem preço” (SNP, Health Has No Price) program, which began in February 2011 granted 100% subsidy (i.e., no patient copayment) under the Farmácia Popular program for medicines used to treat diabetes and hypertension [23]. It was implemented in both public and private pharmacies that were already enrolled in the FPP or AFP under the previous policies. In June 2012, medicines indicated for asthma treatment were also included [27].

The number of pharmacies under the programs has increased over time (Table 2). In the public sector FPP, the largest growth was between the implementation in 2004 (27 facilities) and 2009 (530 facilities), when investment in developing additional public pharmacies stopped. The private sector AFP has a greater number of pharmacy outlets compared with the public program. It started with 2955 accredited facilities in 2006 and by the end of the study period (2013), the number was over 25,000 (Table 2).

Considering coverage in terms of the number of municipalities with at least one participating private pharmacy, the AFP began with 7% of overall national coverage; this rate was higher in the Southeast (13.2%) and lower in the North (2.2%) regions, and concentrated in the medium/large municipalities all regions (Table 3). After initiation of AFP Phase II in April 2009, coverage grew rapidly to 40.5% of municipalities, or six times greater than in 2006. This growth was intensified by the 2011 SNP policy which made medicines for diabetes and hypertension available free of charge in all regions. By 2012, the AFP program covered 63.4% of municipalities, nine times the coverage in 2006; however, despite recent growth in the North (Growth Index=13.1) and Northeast (16.8), coverage remains less than 45% in these regions (Table 3). Municipalities under 20,000 inhabitants present a Growth Index of 78.9, having started with very low coverage (>1.0%) and achieving 54.6% coverage by 2012.

Considering the number of participating facilities available per 100,000 inhabitants (Table 4), important differences have emerged over time, especially following the 2009 and 2011 program changes. In the public sector, the numbers of participating pharmacies increased after 2006, stabilizing in 2009. The Growth Index was around two throughout the period analyzed with small variations among the regions. In the private sector, the program started in 2006, but in 2009 and 2011, there were substantial increases in participation. After the 2009 administrative changes, the AFP program grew rapidly, especially in small municipalities (Table 4); however, penetration of the program remains higher in the South and Southeast.

**Table 4:** **Number of** ***“Farmácia Popular”*** **Program facilities per 100,000 inhabitants and Growth Index by public and private sector and by small (20,000 inhabitants and under) and medium/large (greater than 20,000 inhabitants) municipalities, Brazil and regions, 2006 to 2012**

	**2006**	**2008**		**2010**		**2012**	
	**No.Per100k**	**No.Per100k (Growth index)**		**No.Per100k (Growth index)**		**No.Per100k (Growth index)**	
**Public pharmacies**							
Brazil	0.14	0.27	(1.93)	0.28	(2.00)	0.29	(2.07)
North	0.16	0.45	(2.81)	0.48	(3.00)	0.45	(2.81)
Northeast	0.18	0.33	(1.83)	0.34	(1.89)	0.35	(1.94)
Southeast	0.13	0.22	(1.69)	0.24	(1.85)	0.23	(1.77)
South	0.13	0.22	(1.69)	0.23	(1.77)	0.24	(1.85)
West-Center	0.09	0.21	(2.33)	0.23	(2.56)	0.22	(2.44)
**Private pharmacies**							
Brazil	1.62	3.47	(2.14)	7.34	(4.53)	12.87	(7.94)
North	0.62	0.90	(1.45)	2.26	(3.65)	4.19	(6.76)
Northeast	0.69	1.19	(1.72)	1.93	(2.80)	5.09	(7.38)
Southeast	2.20	5.92	(2.69)	14.08	(6.40)	24.50	(11.14)
South	2.27	4.70	(2.07)	9.63	(4.24)	15.14	(6.67)
West-Center	1.44	3.04	(2.11)	7.22	(5.01)	16.59	(11.52)
**Private pharmacies by municipality size**							
**Medium/large municipalities (over 20,000 inhabitants)**							
Brazil	1.36	3.16	(2.32)	6.48	(4.76)	10.78	(7.93)
North	0.76	0.99	(1.30)	2.19	(2.88)	4.96	(6.53)
Northeast	0.75	1.31	(1.75)	2.04	(2.71)	5.04	(6.72)
Southeast	1.81	4.00	(2.21)	8.05	(4.45)	11.97	(6.61)
South	1.50	4.89	(3.26)	11.53	(7.69)	18.66	(12.44)
West-Center	0.79	2.74	(3.47)	6.05	(7.66)	13.39	(16.95)
**Private pharmacies by municipality size**							
**Small municipalities (20,000 inhabitants and under)**							
Brazil	0.15	1.87	(12.47)	10.17	(67.80)	21.52	(143.47)
North	0.00	0.15	NA	2.55	(16.77)	10.68	(70.25)
Northeast	0.00	0.19	NA	1.20	(6.20)	8.14	(42.16)
Southeast	0.21	2.83	(13.50)	14.44	(68.78)	25.20	(120.01)
South	0.25	2.59	(10.36)	14.38	(57.52)	29.53	(118.11)
West-Center	0.00	0.35	NA	3.97	(11.29)	18.07	(51.35)

**Figure 2:**
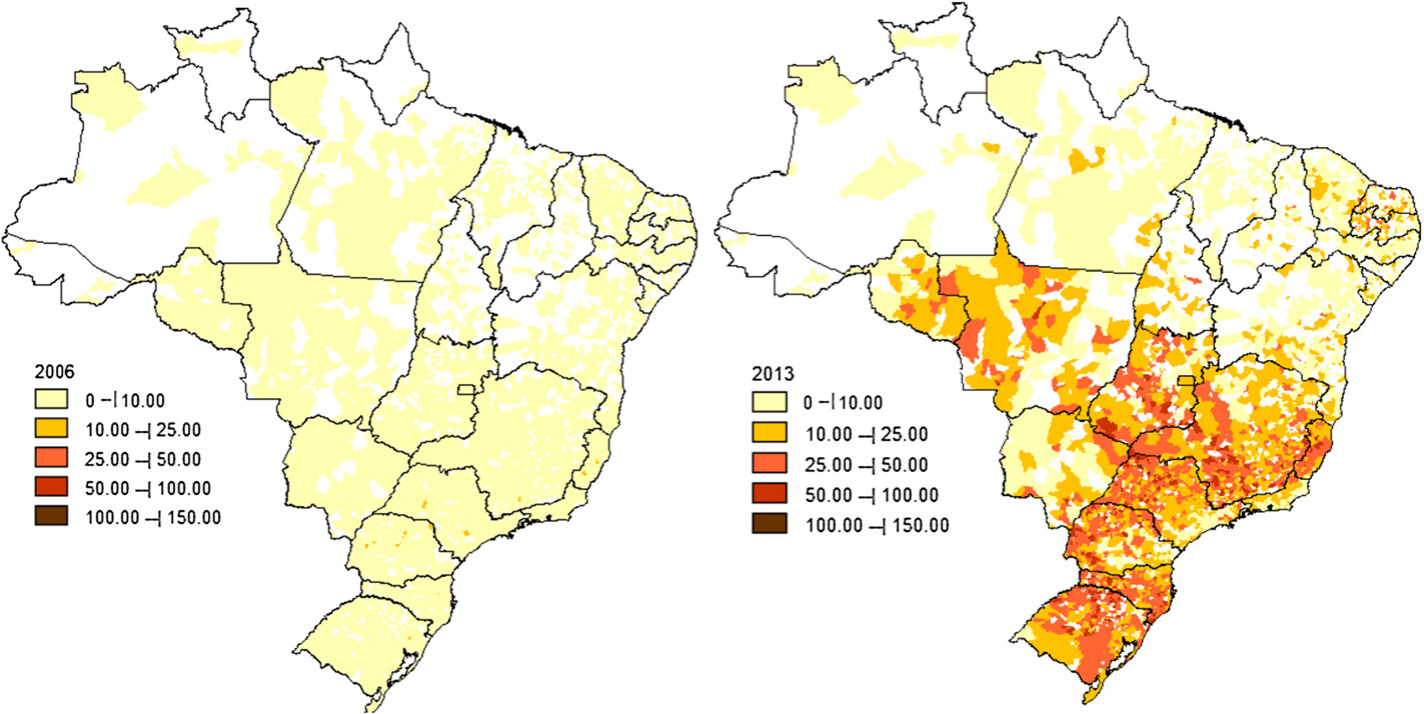
***“Farmácia Popular”*** **coverage in terms of number of pharmacies per 100,000 inhabitants by municipality, 2006 and 2013.**

The thematic maps (Figure 2) illustrate the changes in the Farmácia Popular Program coverage between 2006 when the program began and 2013, the last year of data available. There has been substantial increase in the number of participating pharmacies, which has enormously improved geographic access to medicines through this program.

## Discussion and conclusions

Farmácia Popular is an important innovation in Brazilian public medicines financing. Previous government financing models did not include patient copayments, with medicines provided free-of-charge in public health care facilities. Stakeholders in the national policy process have long argued the advantages and disadvantages of introducing patient copayments, in light of other access to medicines initiatives [28]. To understand the extent to which the Farmácia Popular has achieved its goals of improving access to medicines for its target population and contributed to better health status, it is important to produce empirical evidence that can enlighten this debate.

It is estimated that 90% of the population uses SUS to access health care, but 61.5% people use SUS and other private services and 8.7% did not use SUS [29]. Nevertheless data from PNAD National household Survey) pointed that the Unified Health System (SUS) was responsible for 56.7% of all healthcare, being 61.3 of outpatient medical consultations in 2008 [30].

Utilization depends on household affordability and type of health care needed; According to PNAD 2003 and 2008, there was a reduction in SUS health services utilization associate to an increase of education and income level. In the 1rst quintile of income 88.5% are SUS users, only 20.2% are SUS users in the 5th quintile [30]. The poorest segment of the population always use SUS, while wealthier individuals are likely to use the public sector only for hospitalization or other high cost care. Farmacia Popular Program targets diseases that are sensitive to primary health care as well as low-income patients, but with some ability to pay for medicines. In this sense, it expected that by increasing the Farmacia Popular coverage would increase the access to medicines for non-SUS users [31]. Unfortunately, there was not information from scientific literature describing the profile of *Farmacia Popular* users in terms of source of health care after AFP intervention. Previous data showed an important proportion of prescription from SUS in FFP [31].

The increase of *Farmacia Popular* Program coverage in a relatively short period was only possible through a partnership with the private sector in 2006 and with continuous government investments over time on retail pharmacies, which increased the coverage capacity and geographic distribution of the program. Despite its growth, there must be a stronger consideration of equity in the next stages of FPP expansion, in order to not continue the geographic inequalities observed [31,32]. Although an geographic disparity, with a greater coverage in wealthier geographic regions, it was reducing overtime and this inequality does seem to be so marked as the one reported in South Africa [33], where two provinces met the 1 per 10 000 benchmark for community pharmacies.

In addition, the financial sustainability of the program in the private sector should be considered [28], especially in light of the parallel program in public SUS health facilities, since the related dispensing costs are lower in the public sector and the coverage is higher [28,34].

The rapid grow of the *“Farmácia Popular”* Program in the private sector after the Health has no Price program was implemented in 2011, which provided zero copayment for important categories of medicines, is probably due to low efficiency of the public facilities in terms of medicines availability and quality of pharmaceutical services, including waiting times in the pharmacy [35,36].

Participation in large and mid-size municipalities has grown rapidly in the North, although disparities in coverage between regions still exist. Lately, the smaller municipalities have exceeded the larger in terms of participating pharmacies per 100,000 inhabitants. It is important to understand the reasons for differential growth and implement programs to stimulate growth in poorly covered municipalities, states, and regions in order to achieve adequate distribution of pharmacies according to population need. Lately Brazil is incentivizing the expansion of APF in the municipalities included in the “Brazil without Extreme Poverty” (*“Brasil sem miséria”*) [37] in an effort to reduce disparities in the country.

The main limitations of this study include the following aspects. The number of participating pharmacies in a geographic area does not represent the actual number of patients covered, since municipalities with a small population might have a large area, although the two numbers should be correlated. There is no data on the socioeconomic characteristics of patients using FPP services, limiting inferences regarding equity; however, differences in regional socioeconomic status are well known and municipality size differences are a reasonable proxy for urban/rural differences. We have no data on sales volumes by region or municipality size, which might influence decisions by pharmacies to participate in the FPP.

The results indicate that the FPP succeeded in increasing geographic coverage. There have been dramatic increases in FPP coverage in most regions between 2006 when the private pharmacy component started and 2013, but pharmacies remain unequally distributed across geographical regions. Specifically, the wealthy areas in the South and Southeast have higher coverage, with lower coverage mostly in the North and Northeast, areas with the most need for access to medicines, health care, and other basic services such as potable water and sanitization. Increases in program growth in small municipalities indicate some reduction in disparities within the regions. Despite the fact that only a limited number of medications are covered in AFP, the program is an important achievement since the therapeutic categories covered treat highly prevalent diseases.

The growth in the number of participating pharmacies over time has largely mirrored the phases of the FPP. Longitudinal patient-level studies are needed to understand whether these increases in geographic access have been mirrored by improvements in access to and continuity of care, as well as reductions in socioeconomic disparities in access to appropriate pharmaceutical care.

This paper is the first under the ISAUM-Br Project–a study that intend to describe the impact of the subsidize medicine policies in Brazil [17]. Other aspects of the *“Farmacia Popular”* Program such as medicines prices, medicines utilization, proportion of days covered as a proxy of adherence to the program, health care utilization (hospitalizations) will be explore in future research papers.

## Additional files

Additional file 1:
**Complete results of the documents search.**
Additional file 2:
**Farmácia Popular Program – Medicines List.**
