# Supplementary material for: Farmácia Popular Program: changes in geographic accessibility of medicines during ten years of a medicine subsidy policy in Brazil
Source: J Pharm Policy Pract. 2015 Mar 9;8(1):10. doi: 10.1186/s40545-015-0030-x (PMC4403833; doi:10.1186/s40545-015-0030-x)
Supplement: Additional file 2: — Farmácia Popular Program – Medicines List. [file 40545_2015_30_MOESM2_ESM.doc]

| **Medicines/Products** | **FPP (2004)** | **AFP I & II (2006 & 2009)** | **SNP (2011)** |
| --- | --- | --- | --- |
| Acetaminophen 100mg/mL (oral solution) | X¹ |  |  |
| Acetaminophen 200mg/mL (oral solution) | X¹ |  |  |
| Acetaminophen 500mg | X¹ |  |  |
| Acetylsalicylic Acid 100 mg | X¹ |  |  |
| Acetylsalicylic Acid 500 mg | X¹ |  |  |
| Acyclovir 200mg | X¹ |  |  |
| Albendazole 400mg | X¹ |  |  |
| Albumin Human 20% (50mL)* | X² |  |  |
| Albuterol Sulfate 2mg | X¹ | X^5^ | X^7^ |
| Albuterol Sulfate 2mg/5mL (oral) | X¹ | X^5^ | X^7^ |
| Alendronate Sodium 70mg | X¹ | X^5^ |  |
| Allopurinol 100mg | X¹ |  |  |
| Aminophylline 100mg* | X² |  |  |
| Amiodarone 200mg | X¹ |  |  |
| Amitriptyline 25mg | X¹ |  |  |
| Amoxicillin 250mg/5mL | X¹ |  |  |
| Amoxicillin 500mg | X¹ |  |  |
| Ampicillin 500mg | X¹ |  |  |
| Ampicillin 500mg* | X² |  |  |
| Atenolol 25mg | X¹ | X^3^ | X^7^ |
| Azathioprine 50mg | X¹ |  |  |
| Azithromycin 500mg | X¹ |  |  |
| Beclomethasone Dipropionate 200mcg |  | X^5^ | X^7^ |
| Beclomethasone Dipropionate 250mcg |  | X^5^ | X^7^ |
| Beclomethasone Dipropionate 50mcg |  | X^5^ | X^7^ |
| Benzyl Benzoate 2,5% | X¹ |  |  |
| Benzylpenicillin 1200000UI | X¹ |  |  |
| Biperiden 2mg | X² |  |  |
| Budesonide 32mcg |  | X^5^ |  |
| Budesonide 50mcg |  | X^5^ |  |
| Captoprill 25mg | X¹ | X^3^ | X^7^ |
| Carbamazepine 200mg | X¹ |  |  |
| Carbidopa + Levodopa 25mg+250mg | X¹ | X^5^ |  |
| Cephalexin 250mg/5mL | X¹ |  |  |
| Cephalexin 500mg | X¹ |  |  |
| Chlorpromazine 100mg | X¹ |  |  |
| Chlorpromazine 25mg | X¹ |  |  |
| Ciprofloxacin 500mg | X¹ |  |  |
| Clonazepam 2mg | X^6^ |  |  |
| Dexamethasone Topical 0,1% | X¹ |  |  |
| Dexchlorpheniramine 2mg | X¹ |  |  |
| Dexchlorpheniramine 2mg/5mL | X¹ |  |  |
| Dextrose 5% (500mL)* | X² |  |  |
| Diazepam 10mg | X¹ |  |  |
| Diazepam 5mg | X¹ |  |  |
| Digoxin 0,25mg | X¹ |  |  |
| Digoxin 500mg/mL | X¹ |  |  |
| Doxycycline 100mg | X¹ |  |  |
| Enalapril 10mg | X¹ | X^3^ | X^7^ |
| Enalapril 20mg | X¹ |  |  |
| Erythromycin 125mg/5mL | X¹ |  |  |
| Erythromycin 500mg | X¹ |  |  |
| Estradiol/Norethindrone 50mg+5mg/mL | X¹ | X^4^ |  |
| Ethinyl Estradiol/Levonorgestrel 0,03mg + 0,15mg | X¹ | X^4^ |  |
| Ferrous Sulfate 2,5% | X¹ |  |  |
| Ferrous Sulfate 40mg | X¹ |  |  |
| Fluconazole 100mg | X¹ |  |  |
| Fluconazole 150mg | X¹ |  |  |
| Fluoxetine 20mg | X^6^ |  |  |
| Folic Acid 5mg | X¹ |  |  |
| Furosemide 40mg | X¹ |  |  |
| Glibenclamide 5mg | X¹ | X^3^ | X^7^ |
| Haloperidol 1mg | X¹ |  |  |
| Haloperidol 2mg/mL | X¹ |  |  |
| Haloperidol 5mg | X¹ |  |  |
| Hydrochlorothiazide 25mg | X¹ | X^3^ | X^7^ |
| Ibuprofen 300mg | X¹ |  |  |
| Ibuprofen 600mg* | X³ |  |  |
| Insulin Human Isophane (NPH) 100 UI/mL |  | X^3^ | X^7^ |
| Insulin Human Regular |  | X^5^ | X^7^ |
| Ipratropium Bromide 0,02mg |  | X^5^ | X^7^ |
| Ipratropium Bromide 0,25mg |  | X^5^ | X^7^ |
| Isosorbide Dinitrate 10mg | X² |  |  |
| Isosorbide Mononitrate 20mg | X² |  |  |
| Ketoconazole 200mg | X¹ |  |  |
| Levodopa/Benserazide 100mg+25mg |  | X^5^ |  |
| Levonorgestrel 0,75mg | X¹ |  |  |
| Loratadine 10mg | X¹ |  |  |
| Loratadine 10mg | X^6^ |  |  |
| Losartan Potassium 50mg | X^6^ | X^5^ | X^7^ |
| Male Condom | X¹ |  |  |
| Mebendazole 100mg | X¹ |  |  |
| Mebendazole 100mg/5mL | X¹ |  |  |
| Medroxyprogesterone Acetate 150 mg/mL | X¹ | X^4^ |  |
| Metformin 500mg | X¹ | X^3^ | X^7^ |
| Metformin 500mg (extended release) |  | X^5^ | X^7^ |
| Metformin 850mg | X¹ | X^3^ | X^7^ |
| Methyldopa 250mg | X¹ |  |  |
| Methyldopa 500mg | X¹ |  |  |
| Metoclopramide 10mg | X¹ |  |  |
| Metoclopramide 4mg/mL | X¹ |  |  |
| Metronidazole 4% (oral suspension) | X² |  |  |
| Metronidazole (Vaginal) | X² |  |  |
| Metronidazole 200mg/5mL | X¹ |  |  |
| Metronidazole 250mg | X¹ |  |  |
| Metronidazole 500mg/5G | X¹ |  |  |
| Miconazole 2% lotion | X¹ |  |  |
| Miconazole 2% powder | X¹ |  |  |
| N-Butylscopolammonium Bromide 10mg | X¹ |  |  |
| Neomycin + Bacitracin | X¹ |  |  |
| Nifedipine 20mg (Retard) | X¹ |  |  |
| Norethindrone 0,35mg | X¹ | X^4^ |  |
| Nystatin 100000UI/4G Vaginal | X¹ |  |  |
| Nystatin 100000UI/mL Oral | X¹ |  |  |
| Nystatin 25000UI/1G Vaginal | X¹ |  |  |
| Omeprazole 20mg | X¹ |  |  |
| Oral Rehydration Salts | X¹ |  |  |
| Oseltamivir 75mg* | X^5^ | X^5^ |  |
| Penicillin G Benzathine 1200000UI | X² |  |  |
| Penicillin G Potassium + procaine 300000+100000 | X¹ |  |  |
| Penicillin G Potassium 400000UI | X² |  |  |
| Phenobarbital 100mg | X¹ |  |  |
| Phenytoin 100mg | X¹ |  |  |
| Physiologic Solution 0,9% 500mL* | X² |  |  |
| Potassium Chloride 60mg/mL | X¹ |  |  |
| Prednisone 20mg | X¹ |  |  |
| Prednisone 5mg | X¹ |  |  |
| Promethazine 25mg | X¹ |  |  |
| Propranolol 40mg | X¹ | X^3^ | X^7^ |
| Ranitidine 150mg | X¹ |  |  |
| Simvastatin 20mg | X² | X^5^ |  |
| Simvastatin 40mg |  | X^5^ |  |
| Sodium Chloride 0,9% | X¹ |  |  |
| Sulfamethoxazole/Trimethoprim 200 + 40mg/5mL | X¹ |  |  |
| Sulfamethoxazole/Trimethoprim 400mg+80mg | X¹ |  |  |
| Sulfasalazine 500mg | X¹ |  |  |
| Thiabendazole 5% | X¹ |  |  |
| Timolol Maleate 2,5mg |  | X^5^ |  |
| Timolol Maleate 5mg |  | X^5^ |  |
| Valproate Sodium 50mg/mL | X¹ |  |  |
| Verapamil 80mg | X¹ |  |  |

¹ Inclusion in 2004

² Inclusion in 2005

³ Inclusion in 2006

^4^ Inclusion in 2007

^5^Inclusion in 2010

^6^ Inclusion in 2011

^7^ Inclusion in 2012

* Currently no longer available
